# Comparison of Respiratory Pathogens in Children With Lower Respiratory Tract Infections Before and During the COVID-19 Pandemic in Shanghai, China

**DOI:** 10.3389/fped.2022.881224

**Published:** 2022-06-30

**Authors:** Menghua Xu, Pengcheng Liu, Liyun Su, Lingfeng Cao, Huaqing Zhong, Lijuan Lu, Ran Jia, Jin Xu

**Affiliations:** Department of Clinical Laboratory, Children’s Hospital of Fudan University, National Children’s Medical Center, Shanghai, China

**Keywords:** COVID-19, respiratory pathogens, prevalence, children, lower respiratory tract infections

## Abstract

**Objectives:**

This study aimed to assess the impact of COVID-19 on the prevalence of respiratory pathogens among hospitalized children with lower respiratory tract infections (LRTIs) in Shanghai.

**Methods:**

Respiratory specimens were collected from children with LRTIs in Children’s Hospital of Fudan University from February 2019 to January 2021 and common respiratory pathogens were detected using multiplex PCR. The data of 13 respiratory pathogens were analyzed and compared between the year of 2020 (from February 2020 to January 2021) and 2019 (from February 2019 to January 2020).

**Results:**

A total of 1,049 patients were enrolled, including 417 patients in 2019 and 632 patients in 2020. In 2020, 27.53% of patients were tested positive for at least one pathogen, which was significantly lower than that in 2019 (78.66%). The top three pathogens were Mycoplasma pneumoniae (Mp), human adenovirus (ADV) and human rhinovirus (RV) in 2019, whereas RV, human respiratory syncytial virus (RSV) and human parainfluenza virus (PIV) were the predominant ones in 2020. The positive rates of Mp, ADV, RV, PIV, Influenza virus B (InfB), H3N2, and H1N1 were significantly decreased in 2020. RV was the most detectable respiratory pathogen in 2020, and become the most frequent pathogen in all five age groups. PIV had a high prevalence from October to December 2020 which was even higher than that in 2019. Influenza virus A (InfA) was not detected in 2020. Co-infection was significantly less frequent in 2020.

**Conclusion:**

The public health interventions aiming to eliminate COVID-19 have great impact on the prevalence of common respiratory pathogens. The prevalence of RV and PIV reminds us a possible resurgence of some pathogens.

## Introduction

The coronavirus disease 2019 (COVID-19), caused by severe acute respiratory syndrome coronavirus 2 (SARS-CoV-2), emerged in Wuhan China, in December 2019 (1). In Shanghai, the first COVID-19 patient was identified on 20 January 2020. For the substantial growing outbreak, the World Health Organization (WHO) declared a pandemic on 11 March 2020 (2).

Soon after the emergence of community transmission of COVID-19, the Chinese government has introduced progressive border restrictions. The following nationwide non-pharmaceutical interventions (NPIs) were ordered aiming to eliminate COVID-19, such as prohibiting social gatherings, wearing masks, hand hygiene, postponing the spring 2020 semester in primary and middle schools, and even canceling all face-to-face teaching in schools (3). Remote work at home was also recommended for employed people whenever possible. These evidence consisted of global experience and previous pandemics (4). In previous studies, social distancing could reduce the spreading of seasonal influenza in workplaces (5). The real effect of these NPIs on the spreading of diseases remains unknown, but it appeared to effectively eliminate community transmission of COVID-19 in China in 2020 (6). Studies from Australia, New Zealand, Japan, and the United States also observed the decrease of respiratory viruses, especially seasonal influenza and human respiratory syncytial virus (RSV), when compared with previous seasons (7–10).

Lower respiratory tract infections (LRTIs) are one of the most common childhood diseases and a leading cause of morbidity and mortality in children especially those under 5 years old (11). A number of pathogens are capable of causing LRTIs including bacteria, virus, fungi and atypical pathogen, among which viral respiratory tract infection is the leading cause (appropriate 80%) (12). The major viral pathogens include influenza virus, RSV, coronavirus, adenovirus, and rhinovirus. Previous study showed that in children below 5 years, the combined global mortality of only influenza and RSV reaches 300 000 deaths each year (13). *Mycoplasma pneumoniae* (Mp) is also considered a common cause of community-acquired pneumonia. Mp is identified to be responsible for up to 40% of community-acquired pneumonia in children elder than 5 years (14, 15).

However, there were few studies of how the respiratory pathogens and clinical epidemiological features changed during the outbreak of COVID-19 in Shanghai. To evaluate the prevalence of common respiratory pathogens among hospitalized children with LRTIs in Shanghai in 2020, and to compare it with the prevalence patterns in the year of 2019, we explored the changes in epidemiology of the common respiratory pathogens from February 2019 to January 2021.

## Materials and Methods

### Study Subjects and Ethics Statement

In this study, respiratory specimens (nasopharyngeal aspirates/bronchoalveolar lavage fluid) were obtained from 1,049 enrolled children who were treated for LRTIs in Children’s Hospital of Fudan University by trained staff following standard operating procedures, between February 2019 and January 2021. The specimens were immediately transferred to the clinical laboratory for respiratory pathogens detection. The inclusion criteria were (1) children aged younger than 18 years; (2) hospitalization at the Children’s Hospital of Fudan University; (3) diagnosed with LRTIs (presenting with at least one of the following signs/symptoms: fever, cough, sputum, shortness of breath, lung auscultation abnormality (rale or wheeze), tachypnea, and chest pain (16). Demographics and clinical data from enrolled children were obtained from their electronic medical records. The project was approved by the Ethical Committee of Children’s Hospital of Fudan University.

The patients were divided into five age groups: under 28 days of age, 1–12 months of age, 1–3 years of age, 4–6 years of age, and more than 7 years of age.

### Specimen Detection

A SureX^®^ 13 Respiratory Pathogen Multiplex Kit (Health Gene Technologies, Ningbo, China) was used to detect respiratory pathogens in accordance with the recommended protocol including general influenza virus A (InfA), influenza virus A H1N1 (2009) (H1N1), the seasonal influenza virus A H3N2 (H3N2), influenza virus B (InfB), human adenovirus (ADV), human Boca virus (Boca), human rhinovirus (RV), human parainfluenza virus (PIV), human metapneumovirus (MPV), human coronavirus (CoV), human respiratory syncytial virus (RSV), Chlamydia (Ch), and Mycoplasma pneumoniae (Mp) in all the collected specimens.

### Statistical Analysis

Statistical analyses were conducted using SPSS 23.0 software (IBM, New York, United States). Categorical variables were expressed as numbers (%). Continuous variables were expressed as medians (interquartile range). Proportions for categorical variables (detection rates of virus, sex, and age) were compared using the chi-square test. All of the tests were two-tailed, and a value of *P* < *0.05* represented statistical significance.

## Results

During the study period, 1,049 patients who met the inclusion criteria were enrolled, including 417 patients in 2019 (from February 2019 to January 2020) and 632 patients in 2020 (from February 2020 to January 2021). The most common clinical diagnoses were pneumonia, bronchitis and bronchiolitis. The intensive care unit admission rate was 9.11% (38/417) and 5.85% (37/632) in the year of 2019 and 2020, respectively. Among all the enrolled patients, 12 deaths in hospital in 2019 and 8 deaths in hospital in 2020 occurred. No significant difference was observed in sex ratio between the year of 2019 and 2020. The median age of patients in 2020 (2.8 months) was much less than that in 2019 (21.5 months) (Table 1). To be specific, the proportion of children aged 0–28 days (220/632, 34.81% vs. 46/417, 11.03%, *P* = *0.000*) and 1–12 months (230/632, 36.39% vs. 115/417, 27.58%, *P* = *0.003*) in 2020 increased significantly when compared with that in 2019.

**TABLE 1:** Characteristics and incidence of detected respiratory pathogens in hospitalized patients with lower respiratory tract infections (LRTIs) during the year of 2020 (February 2020 to January 2021) compare with the year of 2019 (February 2019 to January 2020).

	2019 (*n* = 417)	2020 (*n* = 632)	*P*-value
**Characteristics**			
Age, median (IQR), m	21.5 (4.4–60.0)	2.8 (0.5–16.2)	0.000
Male sex, n (%)	232 (55.64)	384 (60.76)	0.109
**Pathogens**			
Mp	119 (28.54)	16 (2.53)	0.000
ADV	89 (21.34)	8 (1.27)	0.000
RV	88 (21.10)	82 (12.97)	0.001
PIV	32 (7.67)	23 (3.64)	0.007
RSV	25 (6.00)	25 (3.96)	0.140
InfB	17 (4.08)	1 (0.16)	0.000
H3N2	16 (3.84)	0 (0.00)	0.000
MPV	10 (2.40)	9 (1.42)	0.249
H1N1	6 (1.44)	0 (0.00)	0.004
Ch	6 (1.44)	10 (1.58)	1.000
Boca	5 (1.20)	3 (0.47)	0.277
CoV	4 (0.96)	8 (1.27)	0.772
InfA	1 (0.24)	0 (0.00)	0.398
Co-infection	80 (19.18)	11 (1.74)	0.000
Total	328 (78.66)	174 (27.53)	0.000

In 2020, of 632 patients, 174 (27.53%) tested positive for at least one of the 13 pathogens, which was significantly lower than that in 2019 (328/417, 78.66%) (*P* = *0.000*) (Table 1). The top three pathogens were Mp, ADV and RV in 2019, whereas RV, RSV and PIV were the predominant ones in 2020. The positive rates of Mp, ADV, RV, PIV, InfB, H3N2, and H1N1 were significantly decreased in 2020, when compared with that of 2019. Other pathogens, like RSV, MPV and Boca presented a lower positive rate in 2020 with no statistical difference. InfA, including HIN1 and H3N2, was not detected in 2020. The most common respiratory pathogens that caused intensive care unit admission were ADV (39.47%, 15/38), RV (18.42%, 7/38), PIV (7.89%, 3/38), and Mp (7.89%, 3/38) in the year of 2019, whereas RV (13.51%, 5/37), ADV (5.41%, 2/37), and PIV (5.41%, 2/37) were the predominant ones in the year of 2020. In the death patients in 2019, the most associated pathogens were ADV (41.67%, 5/12), PIV (8.33%, 1/12), and RV (8.33%, 1/12). RSV (12.50%, 1/8) was the only pathogen found in the death patients in 2020.

**FIGURE 1:**
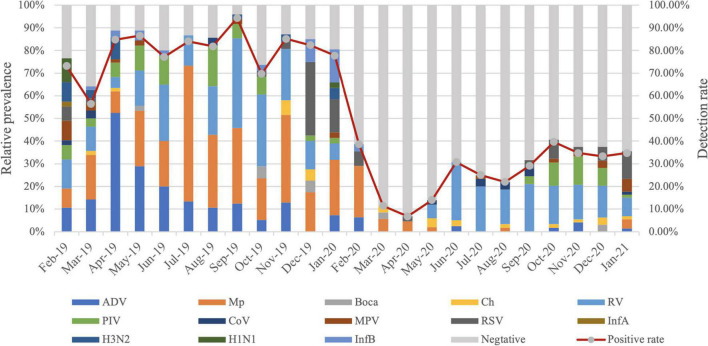
Month-wise distribution of respiratory pathogens detected in hospitalized patients with lower respiratory tract infections (LRTIs) from February 2019 to January 2021.

**FIGURE 2:**
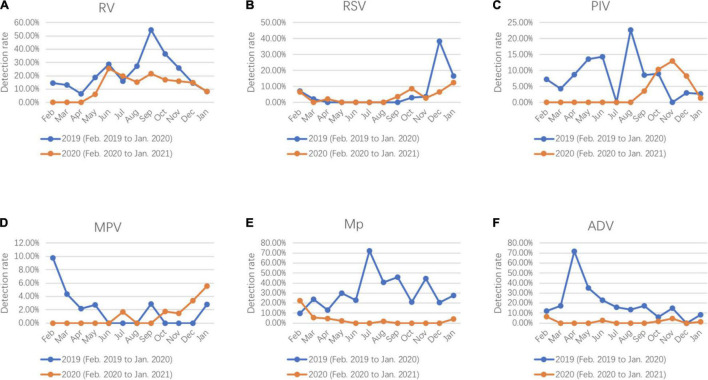
Seasonal activity of respiratory pathogens **(A)** RV, **(B)** RSV, **(C)** PIV, **(D)** MPV, **(E)** Mp, and **(F)** ADV detected in hospitalized patients with lower respiratory tract infections (LRTIs) during the year of 2020 (February 2020 to January 2021) compare with the year of 2019 (February 2019 to January 2020).

Compared with 2019, dramatic reductions were observed for positive rates of the respiratory pathogens after February 2020 (WHO declared public health emergency of international concern) and kept at a lower positive rate (Figure 1). RV was the only respiratory pathogen that remained seasonal distribution pattern with a higher prevalence in June and September. In addition, the positive rate of RV in September 2020 was much lower than that in 2019 (21.82 vs. 54.29%) (Figure 2A). RSV outbreaks occurred in December 2019, but the detection rates in the other months were very low. The detection peak of RSV in 2020 was observed in January 2021 (Figure 2B). PIV had a high prevalence from March to August (spring and summer) in 2019, while it was almost not detected in the same period in 2020. Instead, a high prevalence of PIV was observed from October to December 2020 which was even higher than that in 2019 (Figure 2C). The positive rate of MPV peaked in February in 2019 and in January in 2021, respectively (Figure 2D). Mp was rarely detected in 2020 with a total positive rate of 2.53%. Mp has a clear seasonal distribution pattern as it peaked in July in 2019, and the positive rate of Mp is significantly higher than that in each month of 2020 except February (Figure 2E). We observed that ADV was detected throughout 2019 with a higher prevalence in the first half-year, whereas the phenomenon was not observed in 2020. Instead, ADV is distributed almost evenly throughout the year except for a slight increase in the positive rate in February 2020 (Figure 2F).

**FIGURE 3:**
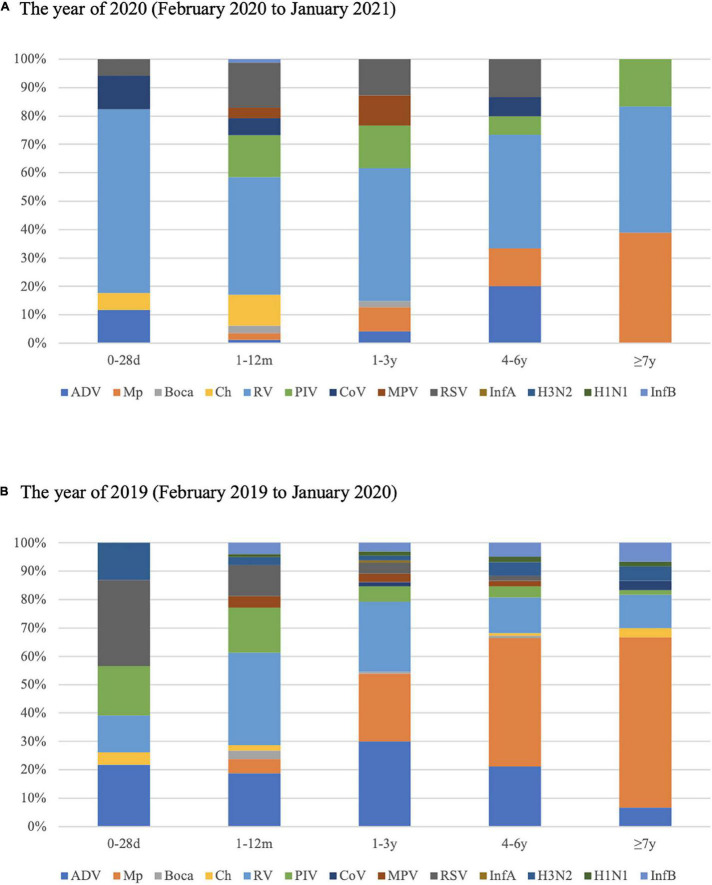
Proportions of respiratory pathogens detected in 2020 (February 2020 to January 2021) **(A)** and 2019 (February 2019 to January 2020) **(B)** according to age group.

The detection rates for respiratory pathogens in all five age groups in 2020 were much lower than that in 2019. The predominant pathogens among different age groups varied between 2019 and 2020. In 2019, for children aged 0–28 days, RSV accounted for 31.82% of pathogens that caused LRTIs, and Mp was not detected. The proportion of RSV decreased with age, and the proportion of Mp increased with age, yet Mp was the most frequent respiratory pathogen in children over 4 years. RV and ADV occupied the majority in 1–12 months and 1–3 years age group in 2019. However, the proportion of RV increased in every age group, and appeared to be the most common respiratory pathogen in all five age groups in 2020 (Figure 3).

Co-infection was still observed in 2020 (11/632, 1.74%), but with significantly less frequency than that in 2019 (80/417, 19.18%). RV was the most frequent pathogen in co-infection in 2020, while Mp ranked the first in 2019 of all mixed infections. Ten triple-infections were found in 2019, whereas no triple-infections was detected in 2020 (Table 2).

**TABLE 2:** Co-infections of respiratory pathogens in hospitalized patients with lower respiratory tract infections (LRTIs) during the year of 2020 (February 2020 to January 2021) compare with the year of 2019 (February 2019 to January 2020).

	2019 (*n* = 417)	2020 (*n* = 632)
**Double infections, n (%)**		
ADV + Mp	15 (3.60)	0 (0.00)
RV + Mp	12 (2.88)	0 (0.00)
ADV + RV	9 (2.16)	0 (0.00)
ADV + PIV	6 (1.44)	2 (0.32)
RV + PIV	4 (0.96)	3 (0.47)
Others	24 (5.76)	6 (0.95)
**Triple infections, n (%)**		
ADV + RV + Mp	4 (0.96)	0 (0.00)
Others	6 (1.44)	0 (0.00)

## Discussion

Studies from many countries have yielded that outpatient and inpatient visits associated with diagnosed infections fell markedly during the COVID-19 pandemic (17–20). These infectious diseases, including LRTIs, are predominantly transmitted through droplets, aerosols, or physical contact which were also believed to be the major transmission routes of COVID-19. In this study, we retrospectively analyzed the prevalence of the most common respiratory pathogens among hospitalized children with LRTIs before and during COVID-19 pandemic. Our study showed that the overall detection rates of respiratory pathogens were 78.66% in 2019 and 27.53% in 2020. It seemed that the series of strict NPIs adopted to contain the COVID-19 pandemic, including wearing masks, hand hygiene, closing schools and social distancing had led to a greatly reduced transmission of LRTIs and affected the prevalence of the common respiratory pathogens.

Interestingly, the proportion of children under 1 year in the number of specimens increased significantly in our study in 2020, thus the median age in 2020 was much less than that in 2019. One possible reason is that the NPIs adopted in the pandemic like closing schools and social distance reduced the outdoor activities of the elder children to a more significant extent. Meanwhile, younger children had poor compliance for the NPIs, as the use of masks, hand washing and the use of gel alcohol before and after contact with other people. Furthermore, for a long time at the beginning of the pandemic, less suitable masks were available for children under 1 year, which also made younger children more vulnerable to respiratory pathogens.

It is known to all, the WHO’s pandemic influenza intervention guidance does not recommend stringent NPIs when pandemic influenza reaches sustained transmission in the population (21). In this study, we have observed an almost absence of the usual winter influenza virus epidemic with a remarkable reduction of InfA, H1N1, H3N2, and InfB during the COVID-19 pandemic, which was also observed in many other areas, including Hong Kong, New Zealand, Finland, Australia, and France (7, 8, 22–24). A previous study found that surgical face masks significantly reduced the detection of influenza virus RNA in respiratory droplets, indicating wearing masks could prevent the transmission of influenza viruses from individuals (25). These results demonstrated that the NPIs, including international mobility restriction, hands-washing, especially masks-wearing could highly reduce the spread of influenza.

In our study, RV was the most detectable respiratory pathogen in 2020, and become the most frequent pathogen in all age groups. Before the re-opening of school (early June, 2020), an absolutely low detection rate of RV was observed. However, after June 2020, there was a sharp increase in the number of detections. Previous studies in Austria, New Zealand, and Japan have also reported a high incidence of RV infection during COVID-19 pandemic (7, 26–28). However to the best of our knowledge, the mechanism that accounts for this phenomenon remains unclear. One possible reason is that RV is a non-enveloped virus, which is relatively resistant to ethanol-containing disinfectant (29) and it can survive on the environment surfaces for a prolonged period of time (30). Additionally, RV infections, are frequently transmitted within households from children to other family members (31). These factors might have contributed to RV infection being less affected by the NPIs.

Remarkably, the prevalence of PIV was basically undetectable from January to August 2020, and an obvious increase in the detections of PIV was observed in the winter season, which were even higher than the same period in 2019. Previous studies showed that PIV plays an important role in hospitalized children with LRTIs with a peak detection in spring and summer, which was similar to the seasonality in 2019 (32). Unlike the previous report, the seasonality of PIV circulation has changed in 2020. The peak detection rate was observed in November 2020. The resurgence was also observed on MPV after October 2020. A study in Shenzhen also observed that the detection rates of PIV and MPV increased from September to December 2020 (33). Except the NPIs, other factors, such as virus competition, the immune response or interference through viral proteins, may also contribute as well. Previous studies have reported that viral interference among influenza virus, rhinovirus, and other respiratory viruses can affect viral infections at the host and population level (34, 35). The lower detection rate of other respiratory pathogens, especially the absence of influenza virus due to the NPIs may partially explain the rise in PIV and MPV. Therefore, we speculate that PIV and MPV should be monitored more diligently in children in the future.

It is reported that the seasonality of RSV has significant changed and a sharp increase in RSV detections occurred at the end of the winter season in Australian in 2020 (26). Another report from New Zealand showed no increase in RSV detections during the whole 2020 winter season (7). Unlike these studies, the detection rate of RSV in our study was much lower in 2020, and the peak detection rate was observed later in January 2021. RSV mostly spreads among the youngest children, however, with the higher proportion of children under 1 year in our study, no higher detection rate of RSV was observed. The mechanism behind this finding is unclear, continued monitoring of RSV epidemic trends and further research are needed.

Like most respiratory pathogens, Mp infection usually occurs during winter months but can happen year-round, that was consistent with the seasonal pattern of Mp in 2019. It is notable that the prevalence of Mp was markedly decreased in 2020 and showed no obvious seasonality. Meanwhile, the detection rate of Mp decreased sharply in all five age groups and the detection rates of Mp in 4–6 years and ≥7 years age groups were as high as 57.31% (47/82) and 59.02% (36/61) in the year of 2019 while they decreased to 5.41% (2/37) and 13.73% (7/51) in the year of 2020. For Mp is transmitted from person to person via respiratory droplets during close contact, outbreaks of Mp infection often occur in military recruits, hospitals, nursing homes, and other long-term care facilities (14, 15). In addition to the NPIs that have been implemented, the chances of children, who are the main reservoirs of Mp, spreading disease in the community may be reduced because they have not been attending childcare facilities and schools during the pandemic. This might partly explained the disappearance of Mp.

An obvious detection peak of ADV was observed in the spring of 2019, which indicated an ADV outbreak in 2019. This was consistent with the results reported by Kong et al., that a small explosive epidemic of adenovirus occurred with a high positive rate in Shanghai from January to May in 2019 (36). Adenovirus-associated severe pneumonia was the leading clinical diagnosis of death in hospital in 2019 which may be related to the ADV outbreak in 2019. However, no detection peak was observed in 2020 with low incidence throughout the year. Because of no consistent seasonal pattern of ADV, further investigation is required to establish the association.

Our study is limited in that all of the data were obtained from one hospital to introduce selection bias. Furthermore, the changes in bacteria were not observed before and during the COVID-19 outbreak. Continued monitoring of viral epidemic trends and further research are needed to help us to better control the epidemic outbreak and correctly handle LRTIs.

## Data Availability Statement

The original contributions presented in this study are included in the article/supplementary material, further inquiries can be directed to the corresponding author.

## Ethics Statement

The studies involving human participants were reviewed and approved by the Ethical Committee of Children’s Hospital of Fudan University. Written informed consent from the participants’ legal guardian/next of kin was not required to participate in this study in accordance with the national legislation and the institutional requirements.

## Author Contributions

MX and JX conceived and designed the experiments and wrote the manuscript. MX, PL, HZ, and LL collected the respiratory samples. MX, LS, and LC performed the experiments and collected the clinical data. MX, PL, LL, and RJ analyzed the data. All authors contributed to the article and approved the submitted version.

## Conflict of Interest

The authors declare that the research was conducted in the absence of any commercial or financial relationships that could be construed as a potential conflict of interest.

## Publisher’s Note

All claims expressed in this article are solely those of the authors and do not necessarily represent those of their affiliated organizations, or those of the publisher, the editors and the reviewers. Any product that may be evaluated in this article, or claim that may be made by its manufacturer, is not guaranteed or endorsed by the publisher.
